# Effect of oleocanthal-rich olive oil on postprandial oxidative stress markers of patients with type 2 diabetes mellitus

**DOI:** 10.29219/fnr.v68.10882

**Published:** 2024-11-11

**Authors:** Maria Efthymia Katsa, Andrea Paola Rojas Gil, Evangelia-Mantelena Makri, Spyridon Papadogiannis, Anastasios Ioannidis, Marianna Kalliostra, Kleopatra Ketselidi, Panagiotis Diamantakos, Eleni Melliou, Prokopios Magiatis, Tzortzis Nomikos

**Affiliations:** 1 Department of Nutrition and Dietetics, School of Health Sciences and Education, Harokopio University of Athens, Athens, Greece; 2 Laboratory of Biology and Biochemistry, Department of Nursing, Faculty of Health Sciences, University of Peloponnese, Tripoli, Greece; 3 Laboratory of Pharmacognosy and Natural Products Chemistry, Department of Pharmacy, National and Ka-podistrian University of Athens, Athens, Greece

**Keywords:** thiobarbituric acid-reactive substances, glutathione peroxidases, red blood cells, protein carbonyls, ibuprofen, platelets, inflammation, postprandial dysmetabolism

## Abstract

**Background:**

Type 2 diabetes mellitus (T2DM) is characterized by postprandial dysmetabolism, which has been linked to post-meal redox disturbances. Oleocanthal (OO), one of the most potent bioactive phenols of extra virgin olive oil, has shown redox modulating properties in vitro. However, its acute, in vivo antioxidant properties have never been studied before.

**Objective:**

The aim of this study was to investigate the kinetics of five redox markers (Thiobarbituric acid-reactive substances [TBARS] and glutathione peroxidase activity in serum-GPx3 and erythrocytes (GPx1), protein carbonyls in serum) after the consumption different meals.

**Design:**

Five different isocaloric meals comprised of white bread and butter (BU) or butter plus ibuprofen (BU-IBU) or olive oil poor in OO or olive oils containing 250 and 500 mg/Kg of oleocanthal (OO250 and OO500, respectively). We hypothesized that OO-rich olive oil will reduce postprandial oxidative stress in T2DM patients compared to other lipid sources. This study involved 10 patients with T2DM and had a cross-over design.

**Results:**

The comparison of incremental Area Under Curves (iAUCs) has shown that OO-rich olive oils were able to alleviate the increments of thiobarbituric acid-reactive substances (TBARS) and GPx3 and induce a higher red blood cells (RBCs) GPx1 activity compared to OO (*P* < 0.05). The effect was dose and redox marker depended. Correlation analysis in the pooled sample demonstrated a positive association between postprandial ex vivo platelet sensitivity to ADP and iAUC TBARS. In conclusion, our study has shown that OO-rich olive oils can favorably modulate lipid peroxidation and RBC GPx activity in T2DM patients when consumed as part of a carbohydrate meal.

**Discussion:**

This study demonstrates for the first time that, apart from its anti-inflammatory and antiplatelet properties, OO can also exert acute antioxidant effects.

**Conclusion:**

This finding emphasizes the health benefits of extra virgin olive oil, particularly those with a high OO content, for T2DM patients.

## Popular scientific summary

- Postprandial oxidative stress is a characteristic feature of diabetes.
- Five redox markers were determined after the consumption of five bread meals by T2DM patients. The meals differed in their lipid source, which was butter, butter/ibuprofen, and olive oil containing 0, 250, and 500 mg/Kg of oleocanthal, respectively.
- Oleocanthal-rich olive oils could favorably modulate lipid peroxidation and erythrocyte glutathione peroxidase activity.
- This is the first study demonstrating acute antioxidant effects of oleocanthal-rich meals in humans.

Diabetes mellitus (DM) is a metabolic disease characterized by disturbed insulin secretion or/and sensitivity leading to chronic hyperglycemia (1). Literature has shown that chronic hyperglycemia is involved in the micro- and macrovascular complications, which often occur in DM (2). The exact pathogenic mechanisms leading to type 2 diabetes mellitus (T2DM) have not been clarified, but oxidative stress seems to contribute to both onset and progression of T2DM (3).

One of the hallmarks of insulin resistance and diabetes is the postprandial dysmetabolism, which is characterized by high increments of glucose- and triglyceride (TG)-rich lipoproteins (chylomicrons and very low density lipoprotein [VLDL]) along with the accumulation of atherogenic lipoprotein particles such as small dense low-density lipoprotein cholesterol (LDL-C) (3). Postprandial hyperglycemia and hyperlipidemia have been linked to acute redox disturbances and increased production of reactive oxygen species (ROS), which cannot be efficiently scavenged in T2DM due to malfunction of the endogenous antioxidant systems of the diabetic patients (4, 5). In addition, postprandial dysmetabolism elevates oxidative stress in pancreatic β-cells, since they have less antioxidant defense enzymes compared to other tissues (6). ROS activates a variety of cellular oxidative stress sensitive pathways, which lead to decreased insulin secretion or even insulin resistance (7). In addition, glucose itself and its oxidative biochemistry causes its auto-oxidation, which further generates ROS and oxidative stress (8). Macronutrients’ intake, in high amounts, is able to promote oxidative stress, contributing also to inflammation through nuclear factor-kappa B-mediated cell signaling pathways (9). Carbohydrates play an important role for the reduction of insulin receptor binding, while they also downregulate the transcription of insulin receptor expression (9, 10). The accumulation of ROS and reactive nitrogen species (RNS) or the reduction of antioxidant capacity in insulin-dependent tissues caused by the increased carbohydrate metabolism possibly changes the phosphorylation procedure of the relevant signaling pathways (9, 11).

Diabetic red blood cells (RBCs) are especially vulnerable to oxidative stress. RBCs allow the transportation of oxygen to peripheral cells via iron (12). As a result, they inevitably come in constant contact with both oxygen and iron, two central molecules in oxidation. Moreover, they undergo non-enzymatic Hemoglobin (Hb) glycosylation, which leads to auto-oxidation of Hb to Methemoglobin (Meth-Hb). Meth-Hb not only lacks the capacity to transport oxygen but also acts as an oxidant itself and attacks membrane lipids, injuring membrane plasticity, harming their abilities to contort as well as reducing their lifespans (13).

Evidence shows that phytochemicals have an important role not only for the maintenance of the mitochondria’s physiologic functions but also for their possible antioxidant and anti-inflammatory properties. There are several phytochemicals, mainly polyphenols, which contribute to the maintenance of redox homeostasis and, as a result, are able to prevent oxidative stress (14, 15). Olive oil is an important and common ingredient of the Mediterranean diet. The phenolic compounds that are contained within extra virgin olive oil (EVOO) act as bioactive molecules with antioxidant properties against the oxidation of lipids, DNA, and LDL-C (16). Among other phenolics hydroxytyrosol derivatives seem to reduce ROS activity, nitrite levels, and COX-2 expression (17). Secoiridoids, especially oleocanthal (OO) and oleacein, inhibit the COX-2 activity (18). In addition, oleacein could inhibit lysine-specific histone demethylase 1A, an epigenetic regulator of metabolic reprogramming in diseases associated with obesity (19). OO, which is the 2-(p-hydroxyphenyl)ethyl ester of (3S)-4-formyl-3-(2-oxoethyl)hex-4-enoic acid and has a molecular weight of 304.34 g/mol (20), has shown to suppress adipocyte inflammation and oxidative stress markers such as Nicotinamide Adenine Dinucleotide Phosphate (NADPH) oxidase and also to enhance the antioxidant capacity through the increase of Superoxide Dismutase (SOD) and Glutathione Peroxidase (GPx) (20).

So far, the majority of interventional studies using EVOOs, rich in polyphenols, were long-term studies, and they usually assessed the redox potential under fasting conditions (21, 22). We have recently shown that an olive oil enriched exclusively with OO was able to exert, in a dose-dependent manner, acute antiplatelet actions in T2DM patients compared to refined olive oil and butter. Its antiplatelet properties were comparable to that of ibuprofen (23). Taking into account the paucity of studies investigating the in vivo antioxidant potential of olive oils rich in OO and the dependence of platelet activity on the redox potential, this study aims to investigate the impact of five meals differing in the lipid source on the postprandial levels of TBARS, glutathione peroxidase activity in serum (GPx3) and erythrocytes (GPx1), and protein carbonyls in serum of T2DM patients. The meals consisted of bread along with butter, butter plus ibuprofen, refined olive oil, and olive oils enriched with two different dosages of OO. This study hypothesizes that OO-rich olive oil will reduce postprandial oxidative stress in T2DM patients compared to other lipid sources.

## Materials and methods

### Study design and participants

This is an acute, postprandial, randomized cross-over pilot study, whose design has already been described in (23). Briefly, patients with T2DM (24) randomly (according to Research Randomizer, randomizer.org) consumed five different meals, with a 2 week gap between them. The carbohydrate meals contained 120 g white bread along with: 1) 40 mL olive oil with low phenolic content (OO), 2) 40 mL of olive oil containing 250 mg/Kg of OO (OO250), 3) 40 mL of olive oil containing 500 mg/Kg of OO (OO500), 4) 39 g butter (BU), and 5) 39 g butter and 400 mg ibuprofen taken half an hour before the consumption of the meal (BU-IBU). Blood samples were collected before meal’s consumption and postprandially. Patients before their participation in the research gave their signed informed consent. This study was in accordance with the Declaration of Helsinki, and it was also been approved by the Ethics Committee of Harokopio University of Athens (ref. number 79548/16-05-2019) and registered to ClinicalTrials.gov (ClinicalTrials.gov Identifier: NCT04419948).

### Participants

In order to be eligible, the participants should be adults (age >18), non-insulin dependent, and having a stable weight (±3 Kg) for the last 2 months. The diagnosis of diabetes was made according to the American Diabetes Association (ADA) criteria (25). Exclusion criteria included anticoagulation or insulin therapy, consumption of food supplements for the last 2 months, the diagnosis of auto-immune diseases, chronic inflammatory disease, cancer, and the uncontrolled thyroid disease. Thirty patients were initially interested in participating in the study; however, volunteers (five males and five females) completed the study. In detail, 10 of them were under insulin therapy and five under anticoagulation therapy, so they had to be excluded. Additionally, five volunteers dropped out of the study. The participants’ medication is shown in Supplementary Table 1.

Participants were given nutritional and physical activity’s guidelines for a period of 3 days before the intervention and then came to the laboratory after a 12 h overnight fast. Specifically, a registered dietician gave guidelines to volunteers in order to avoid of phenolic-rich foods and intense exercise 3 days before the intervention. The suggested diet included a macronutrient composition of 55% carbohydrates, 17% protein, and 28% fat. Participants were also asked to consume the same meal in the evening before each intervention.

### Meals and olive oil characteristics

The meal’s bread was wheat and crustless, specifically ‘Karamolegos Psichatost’, while the butter was ‘Lurpak^®^ Unsalted Butter’. Meals had similar macronutrient composition and caloric content (Supplementary Table 2). Three types of EVOO were used (OO500, OO250, and OO) (23); the OO500 was produced by Kalamon olives variety (Olea europaea L.), which was harvested in November. The malaxation was for 45 min at 30°C, using the OMPHAX SA olive mill’s facilities (23). The Quantitative Nuclear Magnetic Resonance (qNMR), as described by Diamantakos et al., was used in order to measure the phenol content (26). The OO500 was found to contain 500 mg/Kg OO (which means 500 mg oleocanthal/kg of EVOO) and <10 mg/Kg of other common phenolic compounds (oleacein, oleuropein aglycon, ligstroside aglycon, oleokoronal, oleomissional, and tyrosol), while it complied with the extra virgin category (acidity (0.3%), peroxide value [5 meq O_2_], and K indexes [K232 = 1.851, K270 = 0.19]) (23).

The OO was produced by mixing equal volume of OO500 and water at 30°C, followed by mechanical stirring with 30 min duration and centrifugation for separation in the OMPHAX SA olive mill’s facilities (23). After repeating this procedure three times, the content of OO dropped <10 mg/Kg as measured by qNMR, while OO’s lipid profile remained identical with that of OO500 (23). It was found that oleocanthal reacts with water in order to form a hydrosoluble 1,1-diol (oleocanthadiol) (27). Oleocanthadiol, during the centrifugation’s procedure, is able to be transferred from the layer of olive oil to the aqueous layer.

Similarly, the OO250 was produced by mixing equal volume of OO500 and OO, so the OO content, as measured by qNMR, was adjusted at 250 mg/Kg (23). The lipid profile also remained identical with that of OO500 (23).

The meals were prepared every morning before each trial. The bread slices were unpacked from new, sealed bags each time to avoid the presence of moisture. The butter was kept in the refrigerator and was consumed when it reached room temperature. Olive oils were stored in sealed falcon tubes of 50 mL, under nitrogen, at 4°C and came to room temperature under dark. The volunteers were advised to spread the fat on the bread or dip the bread in the olive oil or the melted butter. A registered dietician who was also responsible for the randomization of the study was also responsible for the preparation of the meals.

### Assessment of dietary intake and physical activity habits

The dietary intake of the volunteers regarding the week (before the trial) was assessed by three 24 h dietary recalls. The 24 h dietary recalls were completed the day before the intervention, a random day of the week, and a weekend day (28). The analysis was done with the Nutritionist Pro™ software (version 2.2, Axxya Systems LLC, Stafford, TX, USA). On participants’ first visit, the food frequency questionnaire FFQ (29) was also completed. Physical activity was assessed by using a validated questionnaire (IPAQ-short version) (30).

### Serum, plasma, and RBC isolation from blood

An intravenous catheter was placed in the brachial volunteers’ vein before meals’ consumption. Fasting blood sample was collected in plastic tubes or proper vacutainers for the isolation of serum, plasma, and GPx1 (23). Blood samples were also collected postprandially at 30, 60, 90, 120, 180, and 240 min after each meal’s consumption (23). Glucose, uric acid, and blood lipid profile were measured with Atellica Solution Siemens (23).

The RBC pellet was isolated from EDTA-anticoagulated vacutainers. After the collection of plasma by centrifugation (1,500 × g, 8 min, 4°C), the remaining pellet was washed with saline and re-centrifuged at 200 × g for 8 min at 4°C. After discarding the supernatant, the pellet was washed again with 0.9% saline and centrifuged at 1,500 × g for 10 min at 4°C. The supernatant was discarded, and the remaining RBC pellet was aliquoted in eppendorf tubes and stored at −80°C.

### Anthropometric and clinical markers

Weight, height, waist circumference, body composition, and blood pressure were measured with standard techniques as described in (23).

### Biochemical markers and platelet activity

Glucose, insulin, C-peptide, lipid profile, uric acid, and homocysteine were measured by the biochemical analyzer (Atellica Solution Siemens), while homocysteine was measured by the Immunochemistry analyzer (Centaurus XPT Siemens). All serum analyses were conducted at the same lab following the same procedure (23). Ex vivo platelet aggregation was determined by Light Transmittance Aggregometry as described in (23).

### Redox markers

TBARS is a surrogate marker of malondialdehyde (MDA), which is the end stable product of lipid peroxidation. It is based on the reaction of MDA with thiobarbituric acid yielding a colored adduct (28). The measurement of TBARS in plasma was based on the colorimetric method of Jentzsch et al. (31), adapted for microwell plates. In a mixture of phosphoric acid 0.2 M, butylated hydroxyl toluene 5 μM and thiobarbituric acid 0.11 M 100 μM of plasma were added. After incubation for 60 min at 90°C, cold butanol was added, and the colored adduct was extracted after centrifugation at 12,000 × g for 10 min at 4°C. The butanol phase was transferred in microwell plates, and the absorbance was measured at 532 nm, using a microplate spectrophotometer (BioTek PowerWave XS2). The same procedure was followed for MDA standards of known concentration (0.5–3 MDA μM) produced by acid hydrolysis of 1,1,3,3-tetramethoxypropane as a standard (32). A standard curve was constructed each day, from which the plasma concentration of TBARS was calculated in μM. We also normalized TBARS values for the TG concentration by dividing the concentration of TBARS with the concentration of TGs.

The determination of RBC TBARS is based on the method of Kanias et al. (33). Briefly, using a stock TMP (1,1,3,3-Tetramethoxypropane Malonaldehyde bis) solution of 1 M (in ethanol), standard TMP solutions were generated at 0.2, 0.5, 1, 2, and 5 μM. A total of 400 μL of packed RBCs and standards were mixed at 100:1 with BHT 0.05 M in acetonitrile (Butylated hydroxytoluene) and 2:1 TCA 28% (Trichloroacetic acid) for protein precipitation. After being vortexed and incubated for 10 min at 4°C, all vials were centrifuged at 18°C for 10 min at 15,000 × g. Supernatants were collected in two vials each, for assay and blank measurements. Assay vials were mixed with 4:1 TBA 1% in 0.05M NaOH (Thiobarbituric acid), while blanks were mixed in equal amounts with NaOH only. After vortexing, vials were incubated at 90°C for 60 min and then transferred to 96-well plates for spectrophotometric quantification of TBARS at 532 nm and 453 nm, to correct for interfering substances. Quantities were normalized in g of hemoglobin (Hg) via the protocol described later.

The assay for the Hg determination in RBC has followed the Drabkin and Schmidt principles (34). In short, Hg standards were generated from a stock of a 180 mg/mL bovine Hg solution in Drabkin’s reagent (0.5 mL Brij L23-B4184 in 1,000 mL of deionized water), in concentrations of 0.14 to 0.72 mg/mL. In 96-well plates, standards, blanks (Drabkin’s reagent), and RBC samples in Drabkin’s reagent (2:1,250) were spectrophotometrically quantified at 540 nm after incubation for 30 min at room temperature.

The content of reactive carbonyls in plasma was the key measured factor of oxidized proteins’ accumulation. The Protein carbonyls (PC) assay kit used was Sigma Aldrich. The oxidized proteins in plasma were evaluated in a colorimetrical manner through the process described by the manufacturer. In each sample, 100 μL of 2,4-dinitrophenylhydrazine (DNPH) was added, and then the prosecure followed an addition of 100 μL of 100% TCA solution at –20°C, 500 μL of ice-cold acetone and sonication. A centrifugation at 13,000 × g and an addition of 200 μL of 6 M Guanidine solution and another sonication were the final steps before the measurement of absorbance at 375 nm. The value of oxidized protein had to be normalized to the concentration of total protein, as there was a protein loss observed in the washing steps and had to be considered (32). Specifically, 5 μL of samples were transferred to another set of wells. A protein assay was performed in order to determine the amount of protein per sample. A protein standard curve of bovine standard albumin was generated (32).

The GPx3 activity was determined by the method of Wendel (1980), adapted for microwell plates (35). Briefly, GPx3 catalyzes the reduction of H_2_O_2_ to water using reduced glutathione (GSH) as an electron donor. GSH is converted to its oxidized form (GSSG), which is recycled back to GSH by glutathione reductase using NADPH as an electron donor. The rate of NADPH absorbance reduction is proportional to the GPx3 activity. Various amounts of plasma (10–30 μL) were added to a mixture that contained phosphate-buffered saline 50 mM/EDTA 0.4 mM/pH 7.0/sodium azide 1 mM, GSH reductase (1 U/mL), GSH (1 mM), and NADPH (0.12 mM). After the addition of H_2_O_2_ in the mixture (final concentration 0.0105%, v/v), the absorbance of NADPH was measured at 340 nm and recorded for 7 min, at 1 min intervals in a microplate reader. For the quantification of GPx3 activity, a standard curve was constructed by following the aforementioned procedure with a series of GPx3 standards (4).

For the determination of RBC GPx1 activity, the method of Pleban et al. was used (36). In brief, 50 μL of packed RBCs was mixed 1:5 with icy deionized water, vortexed, and centrifuged at 10,000 rpm for 15 min at 4°C. Supernatants were collected in new vials, further diluted in Drabkin’s reagent 1:5 and vortexed. The supernatans (10–30 μL) were added to the reaction mixture described for the GPx3 assay, and the procedure for the GPx1 activity was similar to that followed for the determination of GPx3. For the GPx1 normalization, Hg was quantified in RBC lysates in deionized water (1:5), as described earlier.

### Statistical analysis

SPSS v.24 (SPSS Inc., Chicago, IL, USA) was used for the statistical analysis, while 0.05 was set as the significance level. The Kolmogorov–Smirnov test was used in order to test the normality of the studied variables. GraphPad Prism 8.4.3 software was used for the AUCs’ calculation. For normally distributed variable, repeated measures ANOVA was used for the comparison during each intervention (ptime, pintervention, and ptime*intervention). The comparisons of baseline values between the studied interventions were made with Independent Samples *t*-test or Mann–Whitney *U* tests. The comparison of the baseline to each time point and between interventions was made with Paired samples *t*-test. Pearson partial correlation coefficients were calculated between normally distributed variables after adjustments for age, gender, body mass index (BMI), and type of meal.

## Results

### Baseline characteristics

Our group of volunteers comprised of five male (aged 54 ± 2.3 years old) and five female (aged 53.6 ± 1.6 years old) Caucasian T2DM patients. All female volunteers were postmenopausal, and none of the volunteers was a smoker. Their baseline, pre-meal, and characteristics did not differ between meals (Table 1 and Figures). No differences were also observed for their dietary intake during the week before each meal (Table 1). The analysis of the FFQs showed that their MedDiet Score was 38.5 ± 0.9, which indicates a satisfactory adhesion to the Mediterranean Diet.

**Table 1:** Baseline anthropometric characteristics, dietary and IPAQ characteristics, and baseline biochemical markers

Studied parameter	Mean ± Std. error					*P*
	Butter (BU) (*N* = 10)	Butter and 400 mg ibuprofen (BU-IBU) (*N* = 10)	Olive oil low in phenolic compounds (OO) (*N* = 10)	Olive oil containing 250 mg/Kg oleocanthal (OO 250) (*N* = 10)	Olive oil containing 500 mg/Kg oleocanthal (OO 500) (*N* = 10)	
Systolic blood pressure/diastolic blood pressure (mmHg)	126 ± 4.2/76 ± 3.3	123 ± 3.7/74 ± 2.6	129 ± 3.2/75 ± 1.7	126 ± 5.1/71 ± 4.1	127 ± 5.5/73.2 ± 3.4	0.937/0.824
BMI (kg/m^2^)	33.8 ± 2.2	33.7 ± 2.1	33.4 ± 2.2	33.8 ± 2.1	33.7 ± 2.2	0.995
Body fat % (according to bioelectrical impedance analysis)	36.6 ± 5	38.8 ± 4.2	38.7 ± 3.9	40.0 ± 4.4	38.3 ± 4.3	0.988
Energy intake (Kcal)	1995.2 ± 179.5	2050 ± 183.7	1915.9 ± 111	2094.4 ± 147.8	2243.5 ± 201.3	0.692
Fat (% of total energy intake)	45.6 ± 3.5	48.7 ± 3.4	49.2 ± 2.7	71.2 ± 17.3	46.1 ± 2.5	0.179
Carbohydrates (% of total energy intake)	40.2 ± 3.4	34.7 ± 4.2	41.3 ± 4.3	40.9 ± 2.6	38.8 ± 2.4	0.695
Protein (% of total energy intake)	13.1 ± 1.2	16.4 ± 1.4	15.1 ± 2	14.0 ± 1.2	14.5 ± 0.9	0.573
Saturated fatty acids, SFA (g)	32.1 ± 4.6	33.1 ± 4.3	28.7 ± 3.5	35.6 ± 4.0	40.6 ± 9.5	0.664
Monounsaturated fatty acids, MUFA (g)	49.7 ± 6.8	52.2 ± 6.3	55.1 ± 4.6	51.4 ± 3	53.5 ± 5.3	0.960
Polyunsaturated fatty acids, PUFA (g)	9.5 ± 0.9	10.2 ± 0.8	11.9 ± 0.9	10.0 ± 1.0	19.7 ± 1.2	0.738
Omega-3 fatty acids (g)	0.8 ± 0.1	0.8 ± 1.9	1.0 ± 0.4	0.7 ± 0.4	0.8 ± 0.1	0.500
Omega-6 fatty acids (g)	6.6 ± 0.6	7.1 ± 0.6	7.2 ± 0.8	6.7 ± 0.5	7.6 ± 0.8	0.872
BMR (Kcal)	1726.3 ± 9.9	1724 ± 87.3	1717.6 ± 95.6	1727 ± 90.9	1723.8 ± 91.9	0.999
Caloric expenditure according to IPAQ (Kcal)	161.1 ± 37.3	195.8 ± 30.4	189.8 ± 35.8	128.7 ± 25.6	151.8 ± 30.9	0.574
Energy balance (Kcal)	-40.7± 200	-75.5 ± 119.8	-138.7 ± 149.9	79.1± 133.7	197.5 ± 200.6	0.633
Glucose (mg/dL)	111 ± 6.0	110 ± 6.0	109 ± 5.3	114 ± 6.8	109 ± 6.6	0.985
HDL-C (mg/dL)	46.5 ± 4.6	45.4 ± 3.6	44.9 ± 3.4	46.8 ± 4.6	46.9 ± 3.4	0.995
LDL-C (mg/dL)	122 ± 16	122 ± 14	124 ± 18	121 ± 16	120 ± 13	0.993
Triglycerides (mg/dL)	168 ± 27	144 ± 14	125 ± 15	132 ± 17	152 ± 20	0.570
Uric Acid (mg/dL)	5.4 ± 0.5	5.6 ± 0.5	5.5 ± 0.5	5.6 ± 0.5	5.5 ± 0.5	0.996

### Effect of meals on the postprandial changes of the metabolic markers and ex vivo platelet aggregation

The effect of the five different meals on the postprandial responses of glucose, insulin, c-peptide, total cholesterol, HDL-C, LDL-C, TGs, homocysteine, uric acid, and ex vivo platelet aggregation induced by adenosine diphosphate (ADP) and Thrombin-Receptor Associated Peptide (TRAP) has been published recently (23). Briefly, no differences in the kinetics of all metabolic markers were observed between meals. On the contrary, a sustained, dose-dependent reduction of platelets’ sensitivity to ADP and TRAP was found after the consumption of the OO meals in comparison to OO or butter meals.

### Effect of meals on plasma TBARS

**Fig. 1:**
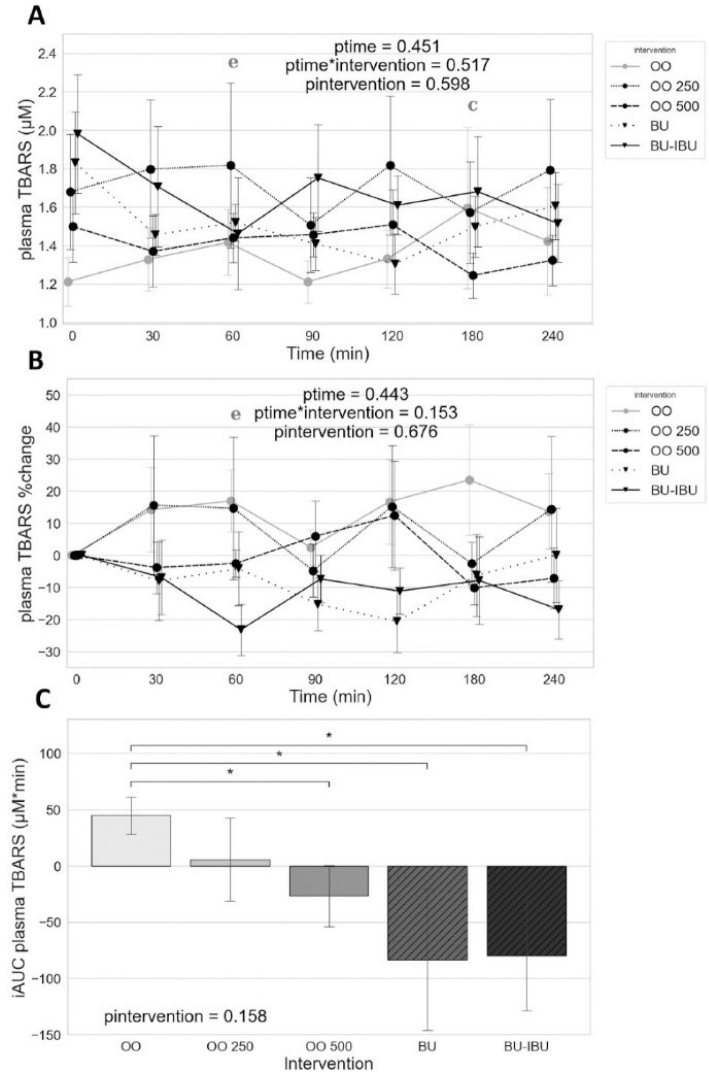
Effect of meals consumption on the TBARS response, measured in plasma. (A) Absolute values, (B) % changes, and (C) iAUCs for the response of TBARS after each meal. BU: butter; BU-IBU: butter and 400 mg ibuprofen; OO: olive oil low in phenolic compounds; OO250: olive oil containing 250 mg/Kg oleocanthal; OO500: olive oil containing 500 mg/Kg oleocanthal. Data were analyzed with ANOVA for repeated measurements, followed by a Bonferroni test for specific time points. Significant differences between post-meal versus pre-meal values (*P* < 0.05) are depicted with the letters (c) OO500 and (e) BU-IBU. Significant difference (*P* < 0.05) between the meals, at the same time point, is depicted with the symbol “*”.

No effect of [time], [time × meal], or [meal] was observed for the postprandial TBARS kinetics. However, the comparison of the incremental Area Under Curves (iAUCs) shows lower levels of TBARS after the consumption of OO500 compared to OO. In addition, the butter meals (BU and BU-IBU) not only did not induce but also kept TBARS levels lower than baseline values resulting in significant differences between the iAUCs of BU, BU-IBU, and OO (Fig. 1).

### Effect of meals on TBARS normalized for triglycerides level

The correlation analysis between fasting TBARS levels and TGs in the total population showed a significant positive correlation between them (*r* = 0.345, *p* = 0.014, *N* = 50). Since the postprandial TGs kinetics may affect the TBARS kinetics, we normalized the TBARS levels according to the TGs levels. Regardless of the type of meal, TBARS/TG ratio declined during the postprandial period because of TGs increase.

**Fig. 2:**
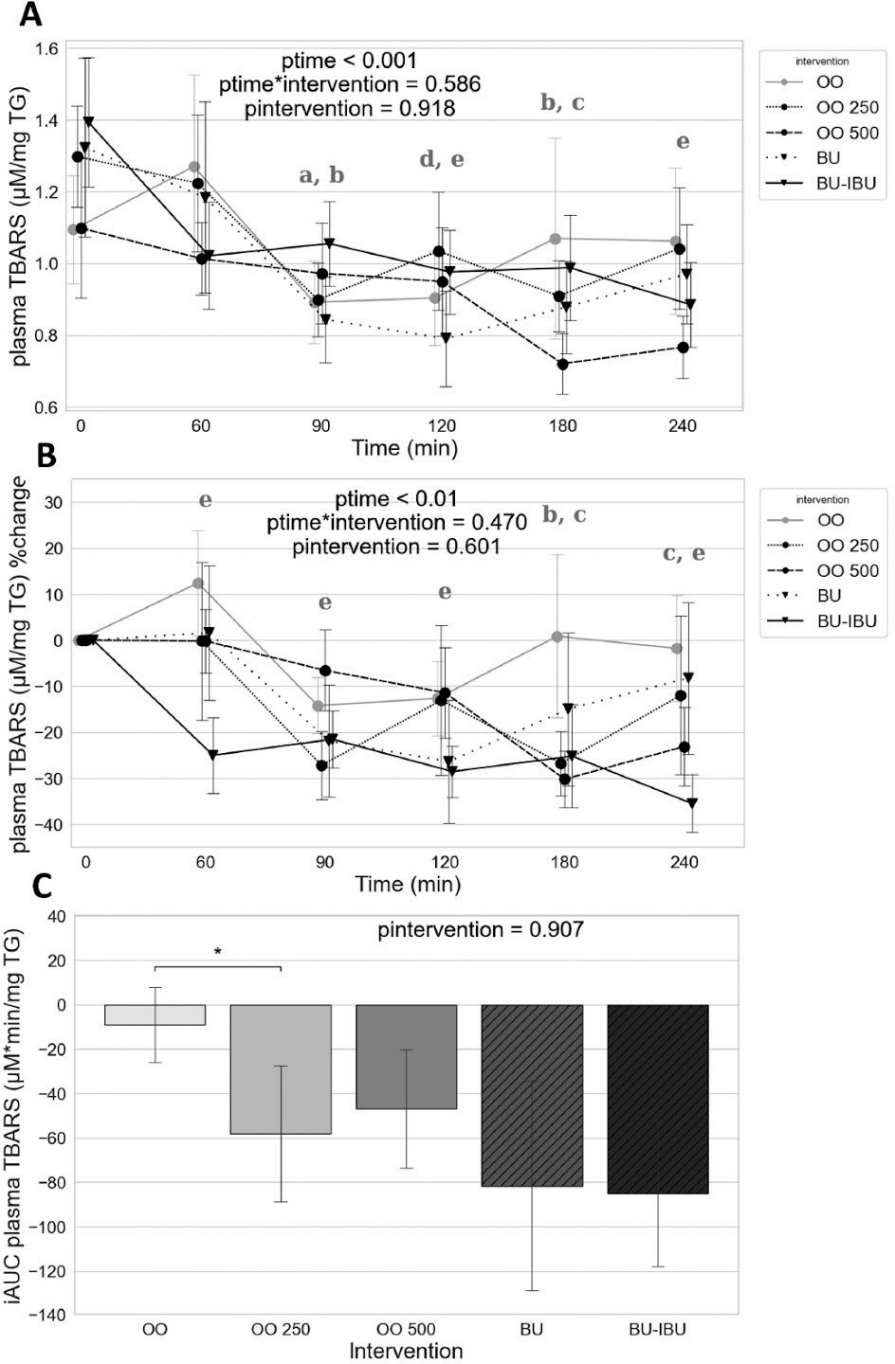
Effect of meals consumption on the normalized TBARS, according to TGs, response. (A) Absolute values, (B) % changes, and (C) iAUCs for the response of normalized TBARS after each meal. BU: butter; BU-IBU: butter and 400 mg ibuprofen; OO: olive oil low in phenolic compounds; OO250: olive oil containing 250 mg/Kg oleocanthal; OO500: olive oil containing 500 mg/Kg oleocanthal. Data were analyzed with ANOVA for repeated measurements, followed by a Bonferroni test for specific time points. Significant differences between post-meal versus pre-meal values (*P* < 0.05) are depicted with the letters (a) OO, (b) OO250, (c) OO500, (d) BU, and (e) BU-IBU. Significant difference (*P* < 0.05) between the studied groups is depicted with the symbol “*”.

A reduction of TBARS/TG was observed at *t* = 90–120 after the OO consumption. While the TBARS/TG levels returned to baseline values after OO, the consumption of OO250 and OO500 kept the TBARS/TG lower than baseline values till 240 min. The same trend was observed for butter meals. The pairwise comparison of the iAUCs has shown a significant reduction of OO250 iAUC compared to OO (Fig. 2).

### Effect of meals on plasma PCs levels

**Fig. 3:**
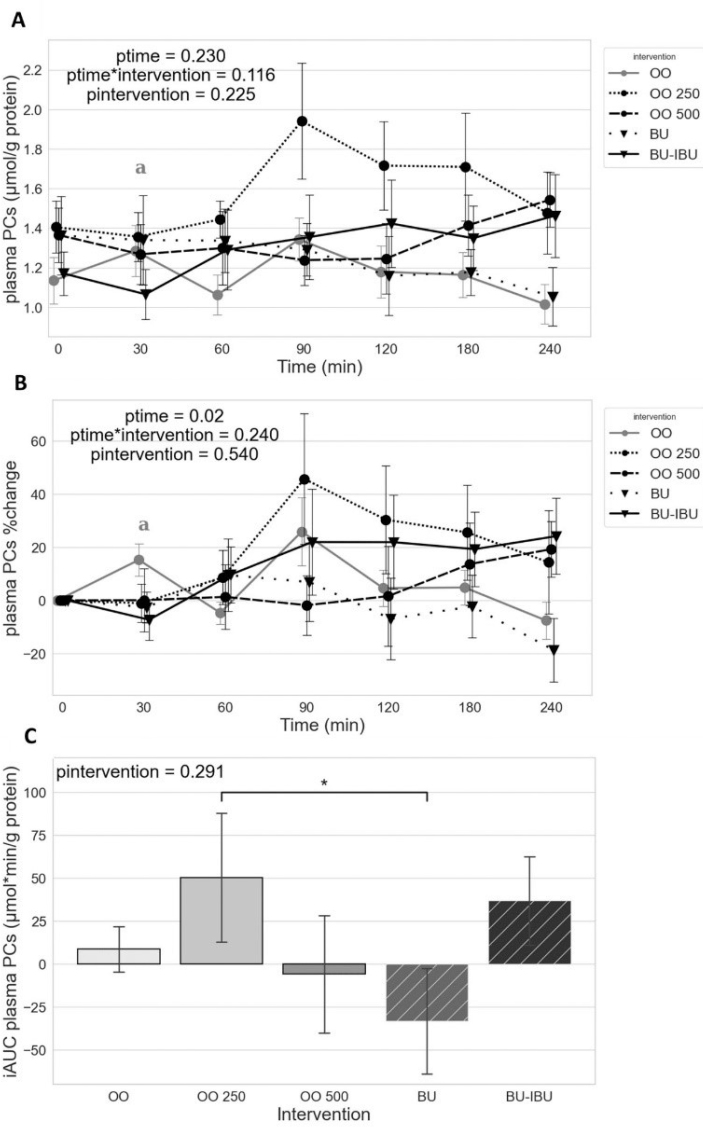
Effect of meals consumption on the PCs response. (A) Absolute values, (B) % changes, and (C) iAUCs for the response of PCs after each meal. BU: butter; BU-IBU: butter and 400 mg ibuprofen; OO: olive oil low in phenolic compounds; OO250: olive oil containing 250 mg/Kg oleocanthal; OO500: olive oil containing 500 mg/Kg oleocanthal. Data were analyzed with ANOVA for repeated measurements, followed by a Bonferroni test for specific time points. Significant differences between post-meal versus pre-meal values (*P* < 0.05) are depicted with the letter (a) OO. Significant difference (*P* < 0.05) between the studied groups is depicted with the symbol “*”.

No significant effect of time, time x group, or group on postprandial PC levels is observed. No significant differences between iAUC were also observed with the exception of a lower iAUC for BU compared to OO250 (Fig. 3).

### Effect of meals on RBC TBARS

**Fig. 4:**
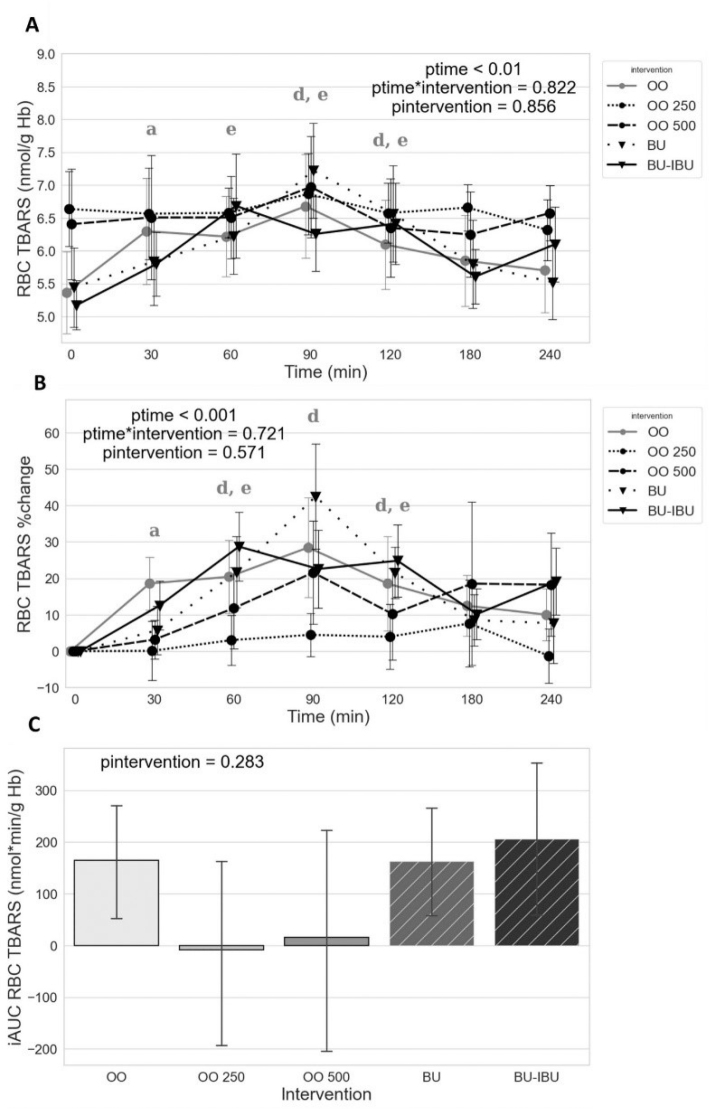
Effect of meals consumption on the TBARS, measured in RBC, response. (A) Absolute values, (B) % changes, and (C) iAUCs for the response of TBARS in RBC after each meal. BU: butter; BU-IBU: butter and 400 mg ibuprofen; OO: olive oil low in phenolic compounds; OO250: olive oil containing 250 mg/Kg oleocanthal; OO500: olive oil containing 500 mg/Kg oleocanthal. Data were analyzed with ANOVA for repeated measurements, followed by a Bonferroni test for specific time points. Significant differences between post-meal versus pre-meal values (*P* < 0.05) are depicted with the letters (a) OO, (d) BU, and (e) BU-IBU.

The butter meals (BU and BU-IBU) and the OO meal induced a similar increase of the RBC TBARS with a peak observed at 60–90 min, depending on the meal. The OO meals (OO250 and OO500, especially the OO250 meal) attenuated the RBC TBARS increases observed postprandially. In addition, lower mean values of iAUC were observed for the OO250 and OO500 meals compared to other meals. However, the large interindividual variation to the RBC TBARS responses prevented this difference from reaching statistical significance (Fig. 4).

### Effect of meals on plasma GPx activity (GPx3)

**Fig. 5:**
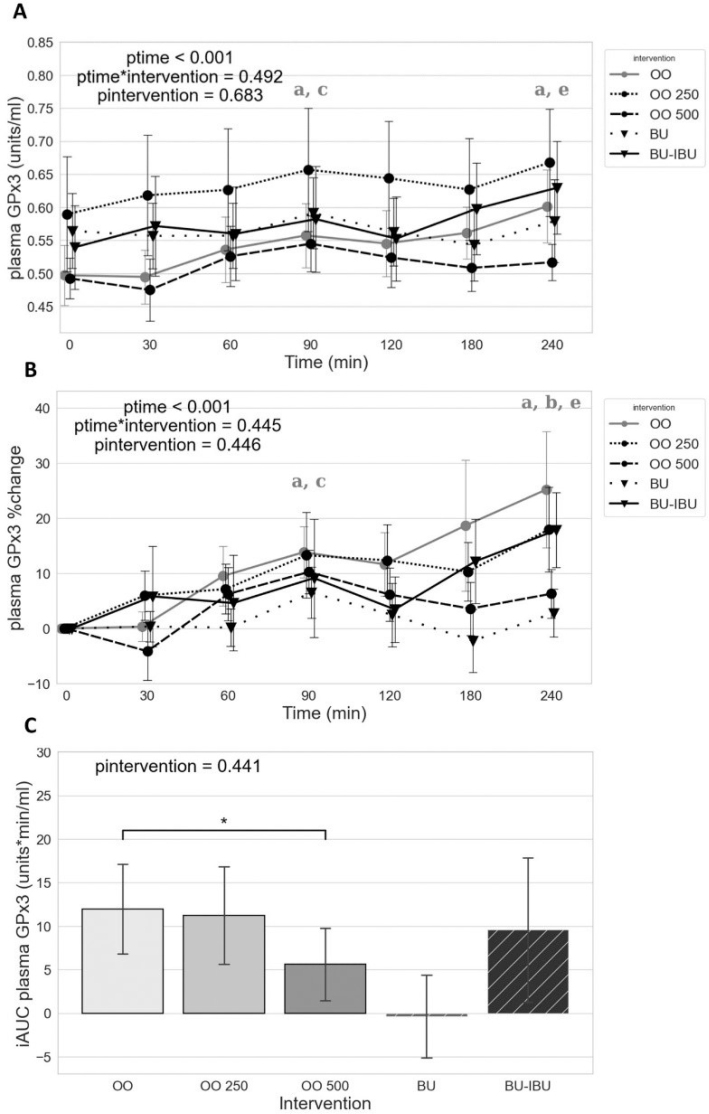
Effect of meals consumption on the GPx3 activity. (A) Absolute values, (B) % changes, and (C) iAUCs for the response of GPx3 after each meal. BU: butter; BU-IBU: butter and 400 mg ibuprofen; OO: olive oil low in phenolic compounds; OO250: olive oil containing 250 mg/Kg oleocanthal; OO500: olive oil containing 500 mg/Kg oleocanthal. Data were analyzed with ANOVA for repeated measurements, followed by a Bonferroni test for specific time points. Significant differences between post-meal versus pre-meal values (*P* < 0.05) are depicted with the letters (a) OO, (b) OO250, (c) OO500, and (e) BU-IBU. Significant difference (*P* < 0.05) between the studied groups is depicted with the symbol “*”.

All olive oil meals induced a sustained increase of GPx3 activity from 60 to 240 min. However, the presence of OO in the olive oils and especially the OO500 was able to attenuate the increase of GPx3 activity. Butter showed a negligible effect on GPx3 activity, and the response of GPx3 activity to the two butter meals was similar (Fig. 5).

### Effect of meals on RBC GPx activity (GPx1)

**Fig. 6:**
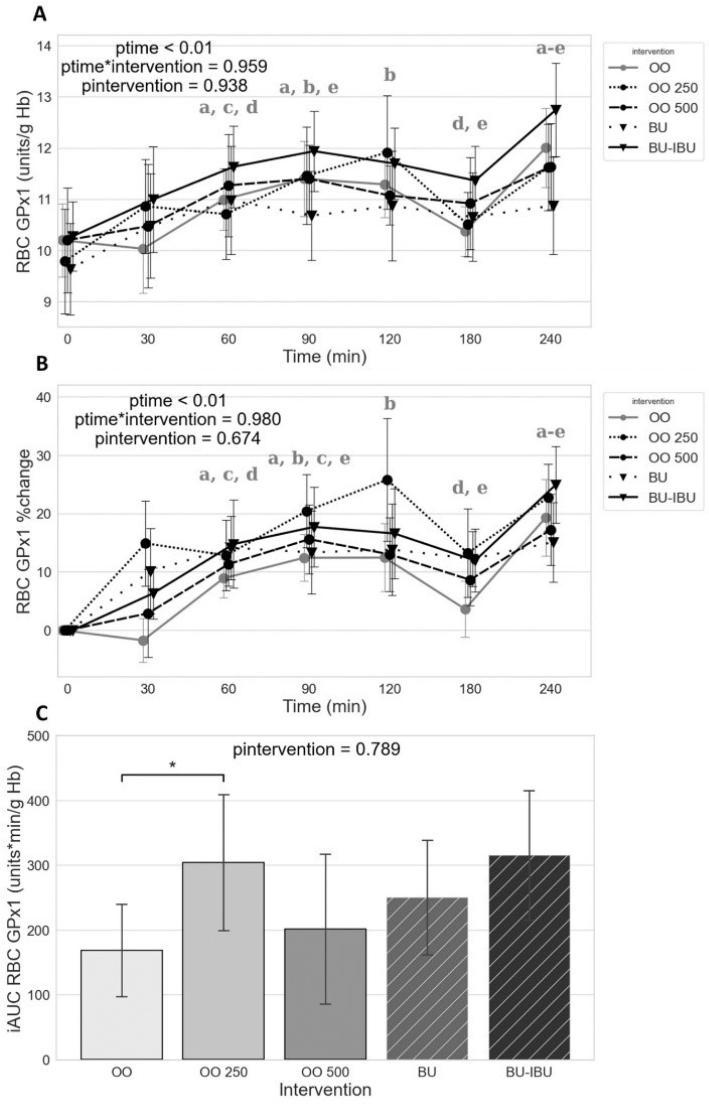
Effect of meals consumption on the GPx1 activity. (A) Absolute values, (B) % changes, and (C) iAUCs for the response of GPx1 after each meal. BU: butter; BU-IBU: butter and 400 mg ibuprofen; OO: olive oil low in phenolic compounds; OO250: olive oil containing 250 mg/Kg oleocanthal; OO500: olive oil containing 500 mg/Kg oleocanthal. Data were analyzed with ANOVA for repeated measurements, followed by a Bonferroni test for specific time points. Significant differences between post-meal versus pre-meal values (*P* < 0.05) are depicted with the letters (a) OO, (b) OO250, (c) OO500, (d) BU, and (e) BU-IBU. Significant difference (*P* < 0.05) between the studied groups is depicted with the symbol “*”.

All meals induced a sustained increase of RBC GPx activity from 30 to 120 min. A transient decline was observed at *t* = 180 min, while at *t* = 240 min, RBC GPx activity increased again. The response of RBC GPx was similar between meals. The comparison of the iAUC levels shows a significantly higher iAUC after OO250 compared to OO (Fig. 6).

Significant difference (*P* < 0.05) between the studied groups is depicted with the symbol “*”.

### Correlation analysis between iAUCs of redox and metabolic and platelet activity indices

**Fig. 7:**
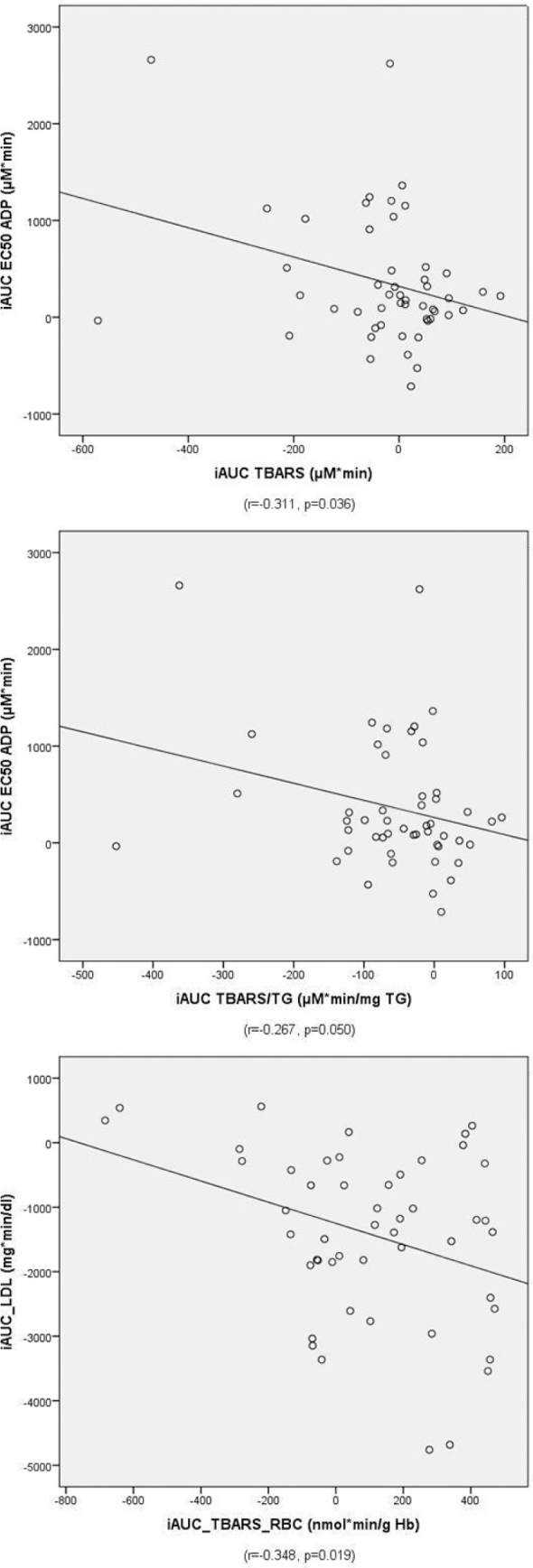
Scatter dot diagrams of the correlation analysis (age, gender, BMI, and type of meal are used as covariates).

We finally proceeded to correlation analysis between the iAUCs of the redox markers and the iAUCs of metabolic and platelet activity indices in the pooled sample. By conducting this analysis, we sought to investigate significant correlation between oxidative stress and postprandial dysmetabolism in an attempt to better understand whether postprandial dysmetabolism can drive postprandial redox responses. Partial correlation analysis using age, gender, BMI, and type of meal as covariates showed significant correlations between iAUC EC50 ADP and iAUC TBARS (*r* = -0.311, *p* = 0.036), iAUC EC50 ADP and iAUC TBARS/TG (*r* = -0.267, *p* = 0.050), and iAUC TBARS RBC and iAUC LDL-C (*r* = -0.348, *p* = 0.019). The scatter dot diagrams of the significant association are shown in Fig. 7.

## Discussion

This study aims to investigate the impact of OO-rich olive oils on TBARS, glutathione peroxidase activity in serum (GPx3) and GPx1, and PC in serum of T2DM patients. For this purpose, our volunteers consumed, in random order, five different meals containing five slices of white bread and 40 mL of three olive oils differing in their OO content or butter with or without ibuprofen intake. Although the primary target of this study was the comparison of the redox response after the consumption of OO-poor and OO-rich olive oils (OO vs. OO250 and OO500), the design of the meals also allowed us to compare the response of the redox profile to meals containing saturated and unsaturated fat (OO vs. BU) but also to assess the redox effects of ibuprofen (BU vs. BU-IBU). In order to estimate the redox profile, we measured markers of lipid peroxidation extracellularly (TBARS, TBARS/TG) and intracellularly (TBARS RBC), extracellular markers of protein oxidation (PCs), and the activity of antioxidant enzymes, namely, glutathione peroxidase, in the circulation (GPx3) and RBCs (RBCs GPx1). The outcomes of this pilot study have shown that the control meals (OO and BU) were able to induce increases of TBARS both in plasma and RBCs and the activation of glutathione peroxidase in plasma and RBCs, too. No effect was observed for PCs. OO-rich olive oils were able to alleviate the increments of TBARS and GPx3 and induce a higher RBC GPx1 activity compared to OO. Correlation analysis in the pooled sample demonstrated an inverse correlation between iAUC EC50 ADP and iAUC TBARS, implying that the higher the postprandial lipid peroxidation, the more sensitive the platelets to ADP, which serves as a physiological endogenous agonist of human platelets.

Postprandial dysmetabolism is a characteristic feature of T2DM resulting from the inability of insulin to properly drive the metabolism of dietary macronutrients or/and lack of insulin due to beta-cell failure (37). In either case, postprandial dysmetabolism leads to excessive and sustained increments of glucose, insulin, and TGs along with the appearance of atherogenic lipoprotein particles such as chylomicron and VLDL remnants (38, 39). Postprandial hyperglycemia and hyper-/dyslipidemia can initiate mechanism of ROS production, which, in combination with the impaired endogenous antioxidant mechanisms of diabetic patients, can lead to postprandial oxidative stress (3, 40). Increased postprandial levels of urinary F_2_-isoprostanes (41, 42), plasma TBARS (43), nitrotyrosine (44), and a decreased bioavailability of NO (45) have been observed in diabetic patients compared to controls. It is therefore obvious that postprandial dysmetabolism is a daily stressor of the redox system for the T2DM patients, leading to sustained increments of ROS after energy-dense meals. In turn, postprandial oxidative stress seems to be one of the main mechanism that mediates the atherogenic potential of T2DM through its deleterious effects on endothelial function (3, 46). Therefore, the presence of antioxidant phytochemicals and vitamins in the meals of diabetic patients may restrain the postprandial ROS increments and increase the nutritional value of the meals. Previous studies have shown that red wine (47), other polyphenols-rich foods (48, 49), and dietary fiber rich foods such as legumes (50) attenuated postprandial redox imbalances induced by fat, glucose, and mixed meal loads.

The antioxidant properties of EVOO have been extensively studied in both animal and clinical studies (16, 51). Therefore, EFSA published several years ago health claim supporting the antioxidant role of OO containing at least 5 mg of hydroxytyrosol and its derivatives per 20 g of OO against LDL-C oxidation (52). OO has been mainly studied for its anti-inflammatory properties, while its antioxidant properties have been inadequately investigated, especially in clinical settings. In vitro, cell studies have shown the ability of OO to exert antiradical activity to ROS such as O_2_●-, HOCl, and ROO●. OO showed better antiradical activity than tyrosol and oleocanthalic acid, but still the in vitro IC50 values were to high (μM-mM) to be biological relevant (53). The amphiphilic nature of OO renders it as an attractive antioxidant molecule for the central nervous system. Indeed, OO could counteract oxidative stress by reducing ROS production and increasing GSH in a H_2_O_2_-induced oxidative stress model in nervous like cells. Proteomic analysis has shown the ability of OO to modulate over 19 proteins. It upregulated proteins related to proteasome, heat shock proteins, and antioxidant enzymes such as peroxiredoxin 1 (53). Apart from its in vitro antiradical activity, OO is able to attenuate the activity of ROS producing enzymes. In an in vitro liver fibrosis model, OO was able to downregulate NOX1,2, and by this way the production of ROS by HepG2 cells (54). OO (100 µM) inhibited NADPH NOX and the production of superoxide anion in isolated human monocytes (55). In addition, both OO and oleacein were shown to suppress TNFalpha induced upregulation of NOX-2 and NOX-4 mRNA in human adipocytes (25 µM). The same study showed that these two phenolic compounds attenuated the TNFalpha induced upregulation of the antioxidant enzymes GPx and SOD (56). Moreover, OO 25–100 µM inhibited the production of ROS and upregulated NRF-2 and HO-1 in LPS-stimulated murine peritoneal macrophages (57). The upregulation of Nrf-2/HO-1 axis was also observed in a murine model of arthritis when dietary OO was able to prevent the collagen-induced rheumatic affection (58). In conclusion, several in vitro studies have shown the ability of OO to favorably modulate oxidative stress by exerting antiradical activities, by upregulating the transcription system of Nrf-2 and the production of antioxidant proteins and by inhibiting the production of ROS through the inhibition of NOXs. However, the dietary OO, during its digestion, is metabolized by phase I (hydration, hydrogenation, and hydroxylation) and phase II enzymes (glucoronidation) (59). Taking into account its poor absorption by the intestine (59), it is obvious that the in vitro antioxidant ability of OO may substantially differ from the ability of dietary achievable amounts of OO to exert in vivo antioxidant effects. Therefore, despite the plethora of evidence supporting the antioxidant role of OO in vitro, there is a need for dietary studies to confirm the ability of OO- or OO-rich olive oils to exert antioxidant protection under clinical or acute postprandial settings.

The novelty of this study is based on olive oils used, the design of the meals, and the redox markers assessed. As far as we are concerned, this is the first study to examine the acute antioxidant effects of OO-rich olive oils in humans. In order to produce an OO-poor olive oil (OO) and two OO enriched olive oils with the same lipid composition, we have chosen a Greek olive oil highly enriched in OO, which was extracted according to the methodology yielding a refined olive oil poor in polyphenols. By mixing the proper amounts of the refined olive oil with the enriched olive oil, we introduced three olive oils differing only in the amount of OO. Taking into account that our volunteers were T2DM, we prepared meals with a high-glucose content in order to induce postprandial hyperglycemia, which, according to the literature, was able to induce redox disturbances, during the postprandial period. We would also like to find out whether the lipid content of olive oil was able to modulate the postprandial redox profile; therefore, we provided a high Saturated fatty acids (SFA), butter meal to our volunteers. We provided an ibuprofen meal as a positive control for the antiplatelet properties of OO, which was the primary outcome of the study (23). Considering the variability of the metabolic phenotype and pharmaceutical treatment of the T2DM patients, we proceeded to a cross-over design minimizing by this way the individual variability in response. Finally, we have chosen to determine a panel of redox indices representing intracellular and extracellular lipid peroxidation, protein carbonylation, and the antioxidant capacity assessed by the activity of extracellular and RBC glutathione peroxidase. The aforementioned indices were determined in seven time points in order to have a detailed valuation of their postprandial kinetics. The use of multiple redox markers in our study provides a comprehensive evaluation analysis of oxidative stress.

TBARS is a surrogate marker of lipid peroxidation, reflecting the end product of lipid peroxidation, MDA. Although HPLC methods for MDA determination are more specific than photometric detection of the MDA-TBA adduct, we chose the faster and less cumbersome TBARS technique to analyze all samples at a lower cost (60, 61). Additionally, we calculated the TBARS/TG ratio since the TBARS concentration correlates with TG levels (*r* = 0.345, *p* = 0.014) pooled baseline samples. Polyunsaturated fatty acids of TGs are the main precursors of lipid peroxidation and MDA in the circulation (60). Therefore, postprandial fluctuations of TGs in TG-rich lipoproteins (chylomicron and VLDL) may strongly impact MDA levels regardless of oxidative stress. We believe that TBARS/TG is a better marker than TBARS alone for assessing the impact of oxidative stress on MDA levels.

Finally, we measured TBARS levels in isolated packed RBCs to reflect the intracellular response of RBCs to lipid peroxidation. As far as we know, this marker has never been measured in postprandial studies before. Despite high interindividual variability, the comparison between iAUCs showed that the OO500 meal resulted in lower increments of plasma TBARS compared to OO. This indicates an acute antioxidant effect of meals containing OO. A similar antioxidant effect was not observed for ibuprofen. Considering the results of our previous study, which showed an acute antiplatelet effect of OO (23), it seems that OO has pleiotropic actions that favorably modulate the postprandial redox and inflammatory responses of diabetic patients.

The in vitro antioxidant properties of OO were first observed in freshly isolated human monocytes from healthy donors, which produced less superoxide anion radical when treated with 100 μM of OO (62). In addition, OO can scavenge ROS, especially HOCl and superoxide anion (53). In vitro studies demonstrated OO’s inhibitory properties against lipid peroxidation in a model of hypoxia-reoxygenation in rat brain tissues (63). Animal studies have also shown that OO-rich diets can attenuate TBARS production in animal models of collagen-induced arthritis in mice (58). However, in most in vitro experiments, the effective doses of OO are not achievable through a human diet, as the effective concentration of OO was much higher than those found in circulation. Our study provides the first data showing that olive oils rich in OO can exert acute in vivo antioxidant properties compared to OO-poor olive oil. This was proven by both the plasma TBARS and the TBARS/TG indices.

In our study, RBC lipid peroxidation tended to be a postprandial event at 30–120 min in all meals that did not include OO polyphenols. Notably, OO250 and OO500 succeeded in reversing that incident and maintained RBC TBARS quite stable throughout the postprandial period. This distinction between plasma and RBCs is reasonable, as RBCs face a higher load of stress. Their storage of Hb naturally predisposes them to oxidation through Hb auto-oxidation by iron (13). Moreover, RBC membranes are permeable to hydrogen peroxide and superoxide, acting as designated free radical scavengers in circulation (64, 65).

Furthermore, RBCs are the highest glucose consumers in the body (66), absorbing glucose through GLUT1 receptors, making their uptake directly dependent on blood glucose concentrations, not insulin (67). Peak glucose concentrations of our volunteers occurred at 90–120 min (23), suggesting high glucose as a key factor in high postprandial RBC redox imbalance. Although mitochondrial postprandial oxidative stress is irrelevant in RBCs, high-glucose-initiated oxidative stress via the polyol and hexosamine pathways, Hb glycosylation leading to auto-oxidation, and membranes rich in polyunsaturated fatty acids (68), as well as potentially scavenging other cells’ mitochondrial oxidative stress (13, 69, 70) render diabetic RBCs especially sensitive to postprandial oxidation. In parallel, plasma MDA and PC studies highlight glucose as the main postprandial oxidative stress generator in obese individuals (71) and diabetic patients as the most prone to oxidation (72).

The levels of PCs are not affected by the meals. A trend for a higher iAUC of PCs after the consumption of OO250 was observed; however, it reached significance only when it compared with the iAUC of BU. High caloric mixed meals can induce either moderate postprandial increase of PC levels (~10–20%) between 90 and 240 min in obese/overweight patients (73) or no effect in diabetic patients (74). Taking into account that the caloric content of those meals was much higher compared to our meals, it seems reasonable that they could not affect postprandial PC levels. Postprandial hyperglycemia seems to be a major determinant of PC levels as shown in a study demonstrating a significant correlation between the iAUC of glucose, from a continuous glucose monitoring system (CGMS) and fasting PCs in patients with impaired glucose regulation and newly T2DM (75). According to our previous paper, postprandial hyperglycemia did not differ between groups, and this may partly explain the similar kinetics of PCs after the consumption of all meals. On the other hand, postprandial hypertriglyceridemia was higher after the OO250 meal, which may also partly explain the higher postprandial PC values after OO250 compared to other meals. The literature lacks mechanistic studies investigating interrelationship between OO and PCs. An animal study has shown that the administration of a high phenolic EVOO to rats, for 14 days, at a dose corresponding to 20 g OO/per day to humans resulted to an almost 20% decrease of their PC levels (76). However, the mechanisms underlying the effect of OO on PCs have not been investigated.

Our study has finally investigated the effect of our meals on the kinetics of the extracellular (GPx3) and intracellular in RBCs (GPx1 RBCs) activity of glutathione peroxidase. GPxs are selenoenzymes able to reduce H_2_O_2_ to H_2_O by using electron donors from reduced GSH (77). GPxs belong to the first line of the antioxidant defense system (78). Our results have shown that all meals apart from BU could activate GPx3, and this activation has shown two peaks at 90 and 240 min. Lower increments of GPx3 activity were observed after the consumption of OO500 compared to OO, implying that OO-rich olive oils could modulate GPx3 activity. A plant-based meal and an energy- and macronutrient-matched conventional meal, with a similar energy content to our meals, increased postprandial GPx3 activity at 120 min in T2DM patients (79). Moreover, an oral fat load could induce upregulation of the GPx1 and GPx4 genes at 4 h in both familial hypercholesterolemia patients and normolipidemic volunteers (80). It is therefore possible that the sustained increase of GPx3 activity can be a result of the upregulation of intracellular GPxs, which could subsequently result to the increased secretion of the extracellular form to the circulation. GPxs synthesis is governed by the action of the Nrf2 transcription factor, which is activated by oxidative stress and xenobiotics (81).

Apart from mixed meals, the effect of Oral Glucose Tolerance Test (OGTT) on GPx activity has been studied in both healthy individuals and patients with metabolic syndrome. A sustained decrease of GPx3 activity between 60 and 120 min was found (78). This effect has also been studied in obese children with impaired or normal glucose tolerance (4). According to this study, children with impaired glucose tolerance had higher GPx3 levels (4). The increase of glucose during OGTT, which was also accompanied by an increase in the antioxidant factors, was characterized as a possible reflection of a compensatory mechanism against the increase of oxidative stress in children with impaired glucose tolerance (4). The lower increments of GPx3 after the OO500 meal may be a result of the attenuated increase of lipid peroxidation due to the antioxidant properties of OO. No study so far has investigated the effect of OO on GPx3 activity; therefore, further studies are required to delineate the direct or indirect mechanisms of by which OO can modulate GPx3 activity.

A sustained increase in RBC GPx activity was observed after the consumption of all meals (90–240 min). It is well known that postprandial hyperglycemia can shift erythrocyte glucose metabolism to the pentose phosphate pathway in an attempt to produce NADPH, which serves as an electron donor for many antioxidant systems (82). This allows GPx1 to face the increased oxidative challenge and the production of H_2_O_2_ due to the increased aerobic oxidation of glucose. However, this mechanism does not explain the sustained increase in RBC GPx activity, especially since in vitro experiments show that RBC GPx1 is reduced under oxidative stress (83). Additionally, the pattern of RBC GPx iAUCs between meals does not correspond to the pattern of RBC TBARS or GPx3, indicating a unique mode of regulation under postprandial conditions. The comparison of RBC GPx iAUC did not show significant differences between meals, except for a significantly higher iAUC after the OO250 meal compared to OO. This effect was not observed for the OO500 meal, indicating a dose-specific effect of OO on RBC GPx activity. One long-term study has shown that increased consumption of EVOO in healthy institutionalized elderly individuals lowers erythrocyte GPx activity (84). However, no acute olive oil or OO studies have been reported in the literature so far.

Finally, correlation analysis between iAUCs in the pooled sample has shown that the higher the postprandial increase in TBARS, the lower the EC50 values for ADP-induced platelet aggregation. Since low EC50 values indicate an increased *ex vivo* aggregatory response of platelets to ADP, this significant correlation suggests that the postprandial sensitivity of platelets to ADP increases with lipid peroxidation. Therefore, the strong antiplatelet activity observed after the consumption of OO-rich meals (23) could be partly attributed to the inhibitory effect of OO-rich olive oils on lipid peroxidation. The activation of platelets by increased oxidative stress in T2DM patients has been proposed as one of the main mechanisms explaining platelet dysfunctionality in these patients (85).

The current study has certain limitations. The small sample size (10 patients) limits the generalizability of the findings. Moreover, there is an heterogeneity regarding the medication received, and we cannot exclude the possibility that this heterogeneity affects the meals’ responses although the cross-over design of the study minimizes this effect.

The postprandial hypertriglyceridemia, in order to be completely assessed, requires longer follow-up (>6 h). Although this study focuses on the acute effects of OO-rich olive oils, it does not address long-term impacts. Nevertheless, daily modulation of postprandial oxidative stress might contribute to a sustained improvement in the redox profile, similar to how managing postprandial hyperglycemia can lead to better glycemic control over time. On the other hand, this study has some important strengths, such as the cross-over design, which minimizes the confounding effects of the different metabolic phenotypes, gender, age, and pharmaceutical treatment of the patients. The OO-rich olive oils had similar fatty acid composition, differing only in the concentration of OO, allowing by this way the assessment of the dose-dependent activity of OO. The inclusion of an ibuprofen meal also allowed us to assess if the antioxidant effects of OO could be attributed to its acute anti-inflammatory or antiplatelet properties. Finally, the wide panel of redox indices measured provides a comprehensive overview of OO’s antioxidant effects.

This study should be considered as a pilot study, which, however, demonstrated promising results concerning the ability of OO-rich olive oils to attenuate postprandial markers of oxidative stress. Of course, this notion should be confirmed in future studies with bigger sample sizes, to less heterogeneous populations for their metabolic phenotype, gender and pharmaceutical treatment. The synergistic effect of OO with other EVOO polyphenols could also be examined in a wide range of concentrations. The redox modulating potential of OO-rich olive oils should be further confirmed with other markers of oxidative stress and wider postprandial periods. Control meals without added fat could also be included where the OO could be administered in the form of a supplement. Ultimately, the antioxidant potential of OO-rich meals should be confirmed with long-term, well-powered, randomized control trials.

In conclusion, our study has shown that OO-rich olive oils can favorably modulate lipid peroxidation and RBC GPx activity in T2DM patients when consumed as part of a carbohydrate meal. This study demonstrates for the first time that, apart from its anti-inflammatory and antiplatelet properties, OO can also exert acute antioxidant effects. This finding emphasizes the health benefits of EVOO, particularly those with a high OO content, for T2DM patients.
